# DECIMER 1.0: deep learning for chemical image recognition using transformers

**DOI:** 10.1186/s13321-021-00538-8

**Published:** 2021-08-17

**Authors:** Kohulan Rajan, Achim Zielesny, Christoph Steinbeck

**Affiliations:** 1 Institute for Inorganic and Analytical Chemistry, Friedrich-Schiller-University Jena, Lessingstr. 8, 07743 Jena, Germany; 2 Institute for Bioinformatics and Chemoinformatics, Westphalian University of Applied Sciences, August-Schmidt-Ring 10, 45665 Recklinghausen, Germany

**Keywords:** Chemical data extraction, Deep learning, Neural networks, Optical chemical structure recognition

## Abstract

The amount of data available on chemical structures and their properties has increased steadily over the past decades. In particular, articles published before the mid-1990 are available only in printed or scanned form. The extraction and storage of data from those articles in a publicly accessible database are desirable, but doing this manually is a slow and error-prone process. In order to extract chemical structure depictions and convert them into a computer-readable format, Optical Chemical Structure Recognition (OCSR) tools were developed where the best performing OCSR tools are mostly rule-based. The DECIMER (Deep lEarning for Chemical ImagE Recognition) project was launched to address the OCSR problem with the latest computational intelligence methods to provide an automated open-source software solution. Various current deep learning approaches were explored to seek a best-fitting solution to the problem. In a preliminary communication, we outlined the prospect of being able to predict SMILES encodings of chemical structure depictions with about 90% accuracy using a dataset of 50–100 million molecules. In this article, the new DECIMER model is presented, a transformer-based network, which can predict SMILES with above 96% accuracy from depictions of chemical structures without stereochemical information and above 89% accuracy for depictions with stereochemical information.

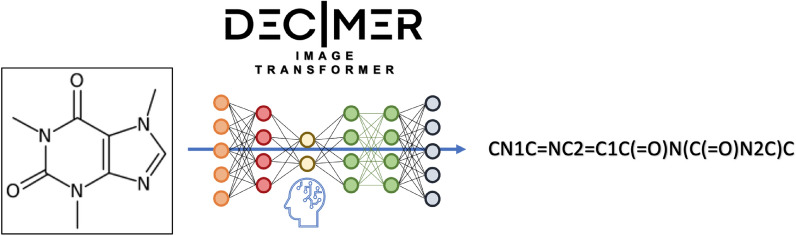

## Introduction

Scientists build on the results of their peers. Knowledge and data arising from previous research is shared through scientific publications and increasingly through the deposition of data in repositories. To enable progress in core areas of chemistry, the availability of open data has a beneficial impact [1]. Most of the chemical data is published in the form of text and images in scientific publications [2]. Retrieving and storing published information into open-access databases will facilitate the reuse as well as the development of new methods and products [3]. But most of the data published is non-machine readable and manual curation is still the standard. This manual work is tedious and error-prone [4]. The increase of publications with valuable chemical information [5] does encourage the development of tools for automated data retrieval. Information retrieval with corresponding database storage is an ongoing task and multiple projects are working towards this. The CHEMDNER [6] challenge is one good example of it.

There has been a significant amount of development in the field of chemical data mining [5] with a couple of open source solutions including ChemDataExtracter [4] and ChemSchematicResolver (CSR) [7], building upon each other. A scanned page of an article, however, cannot be handled by CSR, and not all publications can be processed by CSR. Although most publishers offer documents in markup format, many of the older publications are stored in scanned PDF files. For example, the Journal of Natural Products did publish scientific articles since 1978, one of their issues even dates back to 1949; however, these publications were not formatted in markup format. So retrieving this information is a difficult process.

Image mining methods for chemical structure depictions and their conversion into a machine-readable file format is a comparatively small research area [8]. The automatic recognition of chemical structure depictions and their conversion into machine-readable formats such as SMILES [9] or InChI [10], however, is an important task for creating corresponding databases. The publications include chemical structure depictions along with other information in textual format and contain some information presented as tables, graphs, spectra, etc.

Optical Chemical Structure Recognition (OCSR) software was built to parse chemical structure depictions. However, most of these softwares/tools are unable to handle whole page articles or scanned ones. In order to use these tools, it is necessary to segment the chemical structure depictions into separate images from printed literature and then use these segmented images as inputs. Also, the user should ensure that the image does not contain any other elements or artefacts other than a representation of a chemical structure in a segmented image. All of the available systems vary in their accuracy, OSRA [11] and MolVec [11, 12] can resolve a chemical structure with 80–90% accuracy [8].

With the advancements in computer vision, a few deep learning-based OCSR tools have been developed, e.g. by Staker et al. [13], the first machine learning-based system for segmentation of images and resolution into a computer-readable format. Another deep learning-based work is Chemgrapher [14], where multiple neural networks are combined for the recognition of molecules. Recently, there was a new publication called ChemPix [15], a deep learning-based method that was developed to recognize hand drawn hydrocarbon chemical structures. Another recent publication describes SMILES generation from images [16] where an encoder–decoder method with a pre-trained decoder is used from previous work [17]. These contributions demonstrate an increasing interest in this field of research. Even though they all claim to provide enhanced accuracy, none of them is accessible to the general public to date.

The DECIMER (Deep lEarning for Chemical IMagE Recognition) project [18] is an end-to-end open-source system that can perform chemical structure segmentation on scanned scientific literature and use the segmented structure depictions to convert them into a computer-readable molecular file format.

In our work on DECIMER-Segmentation [19], the segmentation workflow was specifically addressed. Here we now present a transformer-based algorithm that converts the bitmap of a chemical structure depiction into a computer-readable format. The system does not inherit any rules or make any assumptions, thus, it solely relies on the chemical structure depiction to perform its task.

The DECIMER algorithm was primarily inspired by the successful AlphaGo Zero algorithm [20] developed by Google’s DeepMind. The success of AlphaGo Zero allowed us to realize that very challenging problems could be adequately tackled by having a sufficient amount of data and using an adequate neural network architecture. With dozens of millions of molecules available in the databases like PubChem [21], Zinc20 [22], and GDB-17 [23], we have shown in our preliminary communication that our goal to have a system that can work with about 90% accuracy, could be achieved by training the network on a dataset of 50–100 million molecules.

## Materials and methods

DECIMER is a completely data-driven solution to chemical image recognition. Recent impressive applications of deep learning, such as the AlphaGo Zero example, all relied on the availability of very large to unlimited amounts of training data. In our case, one of the largest chemical databases on the planet, PubChem [21], was used.

### Data preparation

The latest version of Pubchem was downloaded from their FTP site. All explicit hydrogens were removed using the CDK [24] and isomeric SMILES [9] were generated, which inherit the canonicalisation and retain the stereochemistry information. After generating the SMILES, the following set of rules were used to filter the dataset for a balanced dataset. The molecules in both training and test set should,

- have a molecular weight of fewer than 1500 Daltons,
- not possess counter ions,
- only contain the elements C, H, O, N, P, S, F, Cl, Br, I, Se and B,
- not contain isotopes of Hydrogens (D, T),
- have 3–40 bonds,
- not contain any charged groups including zwitterionic forms,
- only contain implicit hydrogens, except in functional groups,
- have less than 40 SMILES characters,
- no stereochemistry is allowed.

The resulting main dataset contains 39 million molecules. The same rule set was used to generate a second dataset, but the molecules with charged groups including zwitterionic forms and stereochemistry were retained. Furthermore, the molecules containing tokens that were rare in the dataset were removed (see “Tokenization” section), resulting in a dataset that contains approximately 37 million molecules. Adding extra information caused the SMILES character length to get longer. Later, when the rule that SMILES length should not exceed 40 characters was applied, more molecules were removed. In the end, this resulted in dataset 2 being smaller in size than dataset 1.

Molecular bitmap images were generated using the CDK Structure Diagram Generator (SDG). The CDK depiction generator enables the generation of production-quality 2D images. In this work, every molecule was randomly rotated and depicted as 8 Bit PNG images with a 299 × 299 resolution. It was made sure that each image contains only one structure.

Using the set of images from the second dataset and introducing image augmentations the third dataset was generated. The image augmentations were applied using the imgaug [25] python package. One of the following augmentations was randomly applied to the images.

- Gaussian Blur
- Average Blur
- Additive Gaussian Noise
- Salt and Pepper
- Salt
- Pepper
- Coarse Dropout
- Gamma Contrast
- Sharpen
- Enhance Brightness

**Fig. 1:**
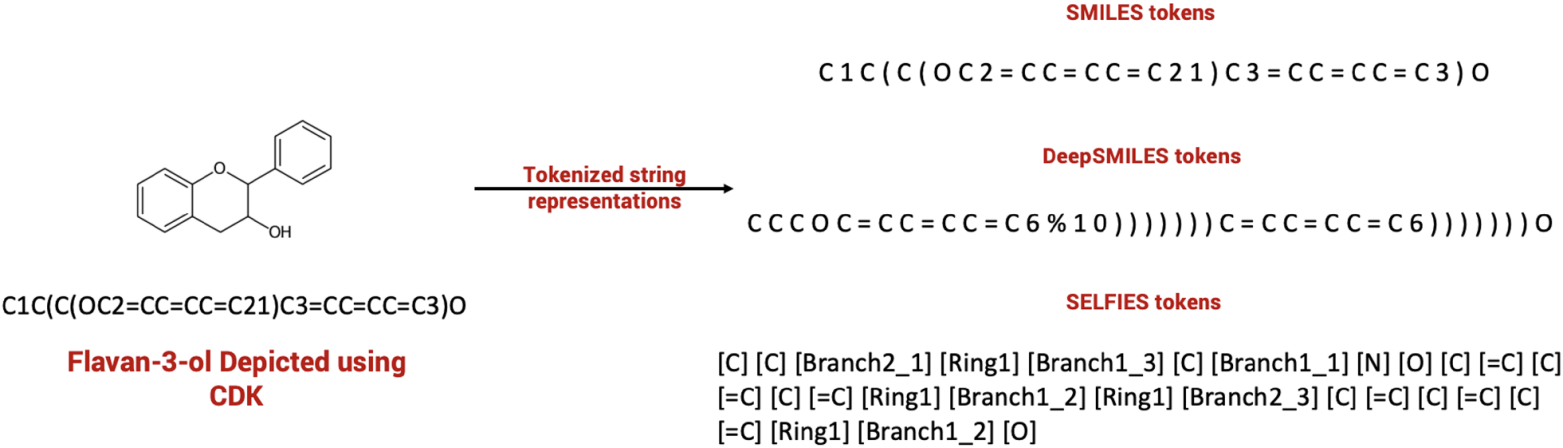
SMILES, DeepSMILES and SELFIES represented as tokens with a space character as a delimiter

Often, deep learning in chemistry is using SMILES as a textual representation of structures. Training Neural Networks (NNs) directly with SMILES, however, has pitfalls: In order to generate tokens, a set of rules has to be set up on how and where to split long strings of SMILES into smaller words. After training, invalid SMILES are often encountered in the predictions, which results in overall significantly reduced accuracy. To tackle this problem there are two new text representations named DeepSMILES [26] and SELFIES [27]. DeepSMILES exhibited better results in comparison to standard SMILES, but again invalid DeepSMILES caused similar problems. In the end, SELFIES were used, since they can be split easily into tokens by splitting the SELFIE at close (“]”) and open brackets (“[”). No further rules had to be applied to split them into a working token set (Fig. 1). Also, they translate back into a SMILES string without any errors. All SMILES strings in our 3 datasets were converted into SELFIES using Python.

To summarize, the datasets used in this work are:

1. Dataset 1: PNG images of chemical structure depictions plus corresponding canonical SMILES converted into SELFIES, without stereochemical information and charged groups.
2. Dataset 2: PNG images of chemical structure depictions plus corresponding canonical SMILES converted into SELFIES, with stereochemical information and charged groups.
3. Dataset 3: Augmented PNG images of chemical structure depictions plus corresponding canonical SMILES converted into SELFIES, with stereochemical information and charged groups.

Test datasets were selected from 10% of each dataset. To ensure that the chemical diversity of test and training data was similar, 10% of SMILES were selected as Test dataset using the RDKIT MaxMin algorithm. An overview of all the train and test datasets and the naming of subsets can be found in Table 1.

**Table 1:** Overview of the datasets

	Dataset 1				Dataset 2		Dataset 3	
Total dataset size	39 million				37 million		37 million	
	Subset 1	Subset 2	Subset 3	Subset 4	Subset 5	Subset 6	Non augmented test set	Augmented test set
Train dataset size	921,600	10,240,000	15,360,000	35,002,240	15,360,000	33,304,320	33,304,320	33,304,320
Test dataset size	102,400	1,024,000	1,536,000	3,929,093	1,536,000	3,700,480	2,000,000	2,000,000

### Image feature extraction

**Fig. 2:**
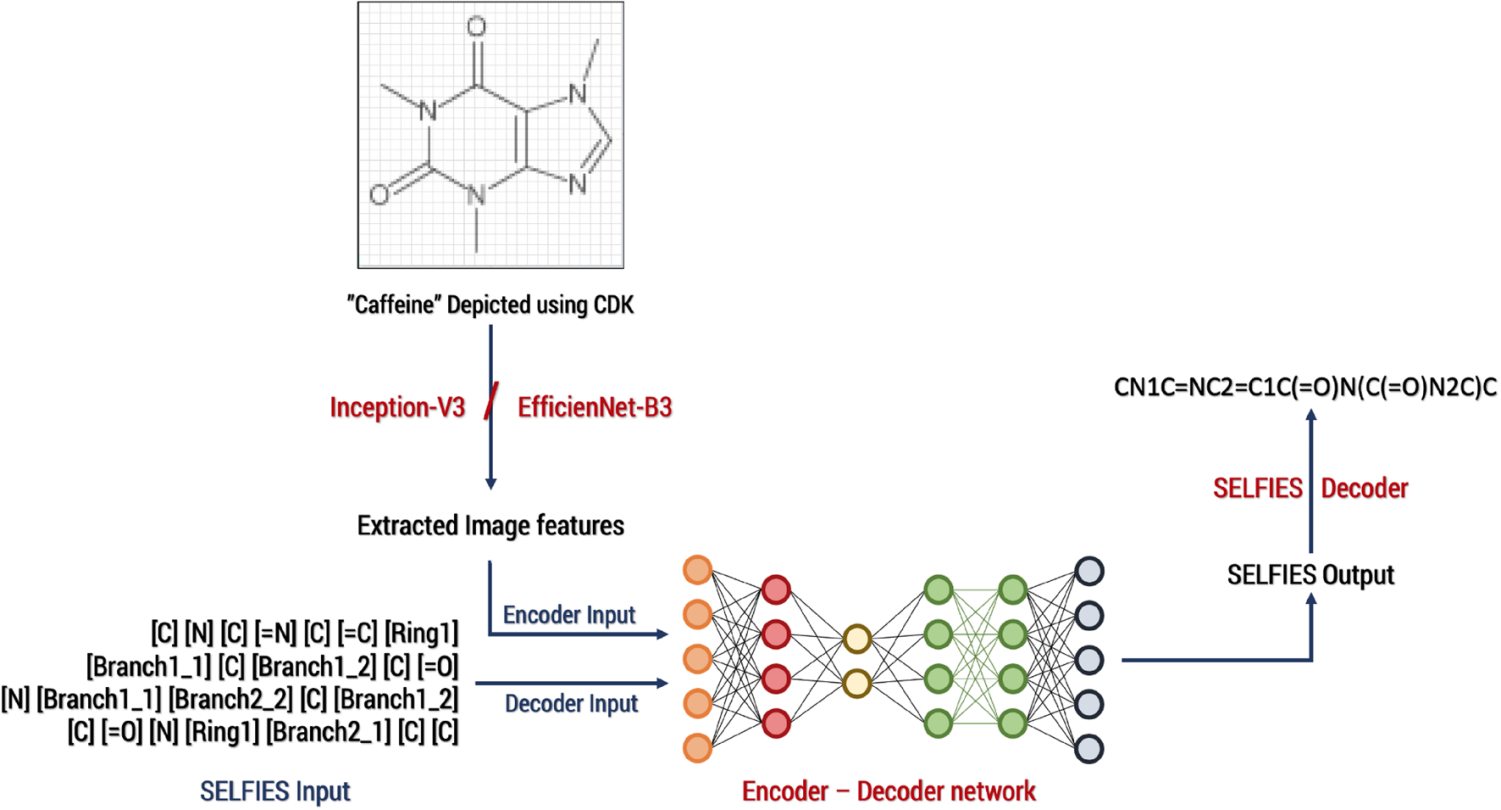
Schema of the encoder–decoder network used in DECIMER for training

A Convolutional Neural Network (CNN) is used to parse the images, where the second last layer retains the features to be extracted for calculations. For training our model, we evaluated InceptionV3 [28] and EfficientNet-B3 [29], see Fig. 2. The EfficientNet base model for an image size of 299 × 299 outperforms InceptionV3 in our task at hand [29].

Every image was scaled to a resolution of 299 × 299 pixels and the pixel values were normalized to interval − 1 to 1, which corresponds to the format used on InceptionV3 and EfficientNet-B3. Then the image features were extracted into a vector format using the pre-trained weights of ImageNet [30] on InceptionV3 and pre-trained weights of Noisy-student [31] training on EfficientNet-B3.

For Inception V3 a feature vector size of 8 × 8 × 2048 and for EfficientNet-B3 a feature vector size of 10 × 10 × 1536 was obtained. The 8 × 8 × 2048 and 10 × 10 × 1536 dimensions are simply the shape of the last output layer in Inception-V3 and EfficientNet-B3 networks. Since Inception-V3 and EfficientNet-B3 are networks built for image classification, these features are then used in the final layer of these networks to classify the images.

Next, these extracted feature vectors were saved into NumPy arrays.

### Tokenization

SELFIES were tokenized into a unique set of tokens and padded to fit the maximum length of SELFIES strings. Here the Keras [32] tokenizer in Tensorflow 2.3 [33] was used. Table 2 summarizes the details regarding the tokens present in each dataset.

**Table 2:** SELFIES tokens and maximum length found on each dataset

Datasets	Number of SELFIES tokens	The maximum length of the SELFIES strings
Dataset 1	27	47
Dataset 2	61	47
Dataset 3	61	47

Tokens in Dataset 1: [C], [=C], [Branch1_1], [N], [Ring1], [O], [Branch1_2], [Expl=Ring1], [=N], [Branch2_1], [Branch1_3], [Ring2], [S], [F], [=O], [Branch2_2], [Cl], [Branch2_3], [#C], [Br], [P], [=S], [I], [=P], [Expl=Ring2], [B], [#N].

Tokens in Dataset 2 & 3: [C], [=C], [Branch1_1], [Branch1_2], [Ring1], [N], [O], [=O], [=N], [Ring2], [Branch2_1], [S], [Branch1_3], [F], [Branch2_2], [Cl], [Branch2_3], [Br], [#C], [/C], [#N], [P], [C@Hexpl], [C@@Hexpl], [=N+expl], [=S], [=N-expl], [I], [O-expl], [N+expl], [\C], [/N], [/O], [C@expl], [B], [C@@expl], [\N], [Expl/Ring1], [\O], [NH+expl], [I-expl], [Expl\Ring1], [P+expl], [NH2+expl], [/Cl], [/S], [NH3+expl], [Cl-expl], [/F], [#N+expl], [C-expl], [\S], [N-expl], [=NH+expl], [=I], [S-expl], [\Cl], [S+expl], [#C-expl], [B-expl], [/Br].

Complete list of rare tokens which were removed: [=B], [=Cl], [=Br], [#I], [=I], [#S], [Expl#Ring1], [#B], [#P], [=Br], [Expl#Ring2].

#### Generating TFRecords

Extracted feature vectors and tokenized data must be converted into TFRecords before training the models on Tensor Processing Units (TPU) [34]. TFRecords stores the data in binary format which allows training the models faster using GPUs and TPUs. The TPUs are currently available through the Google Cloud Platform. TFRecords are stored in a Google cloud bucket for training. This reduces the training time significantly and reduces the overhead of loading the data and performing the calculations on a TPU.

Using a custom python script all the datasets were converted into 75 MB chunks of TFRecords. Each TFRecord contains 128 Datapoints (128 image vectors + 128 tokenized strings).

After generating the TFRecords locally, they were moved to a Google cloud storage bucket.

### Networks

In this work, two different types of networks were evaluated. Initially, an encoder–decoder model was tested, which is based on the work by Google on their *Show, Attend and Tell* [35] publication. The network eventually selected is a transformer-based model based on the *Attention is all you need *[36] publication by Google. The models are written using Python and Tensorflow 2.3 as a backend.

### Encoder–decoder network

The encoder–decoder network used is an unaltered implementation by the TensorFlow team [37]. The model uses a CNN-based encoder with a ReLU activation function, a soft attention mechanism introduced by Bahdanau et al. [38] and the RNN based decoder uses Gated Recurrent Units (GRU) and two fully connected layers. The decoder consists of 1024 units and an embedding size of 512.

The network is trained using an Adam optimizer [39] with a learning rate of 0.0005 throughout all learning epochs. The loss is calculated using sparse categorical cross-entropy between real and predicted SELFIES.

#### Transformer network

**Fig. 3:**
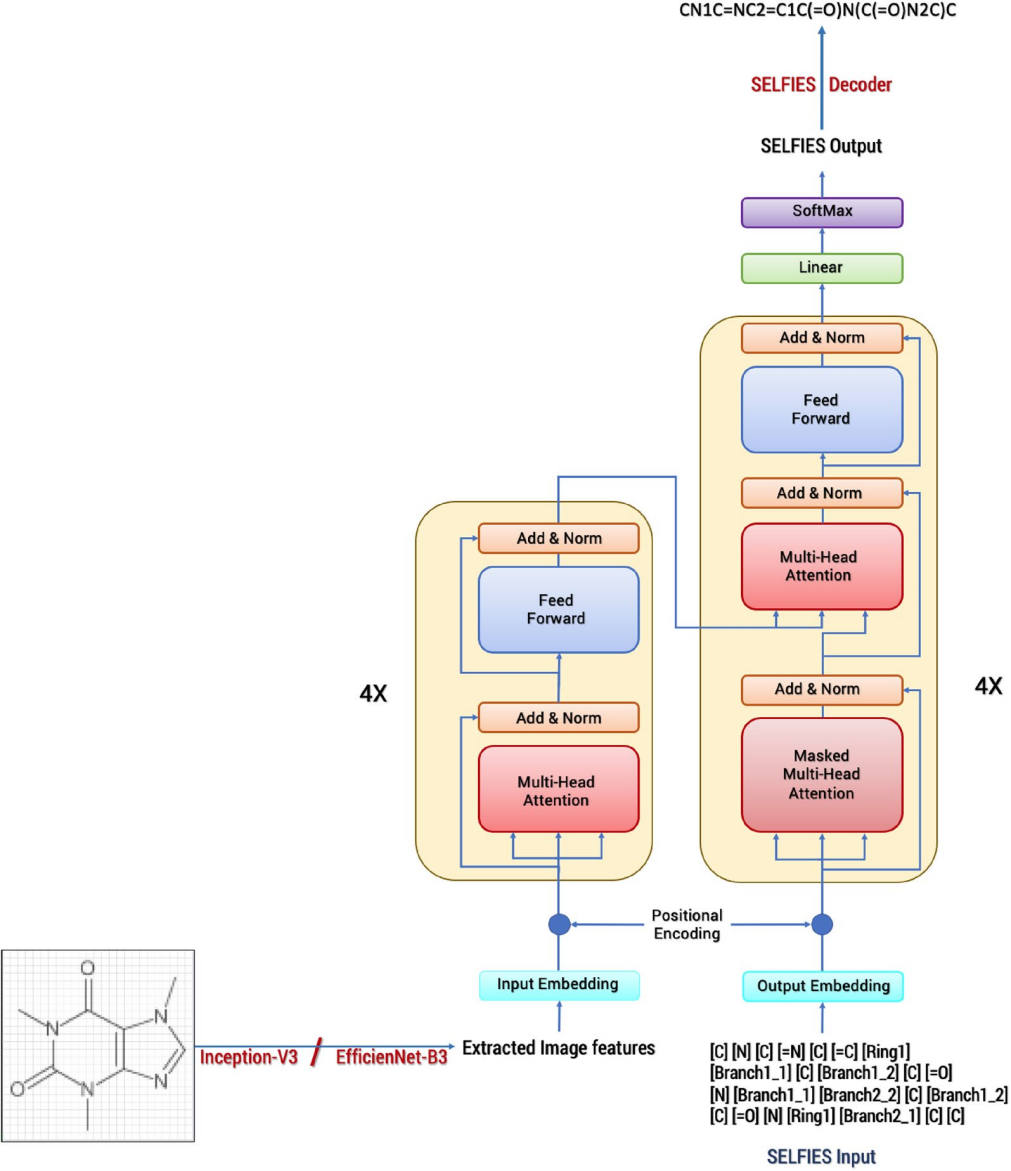
Schema of the transformer network used in DECIMER for training

The transformer model (Fig. 3) used in this work is the model from the 2017 publication *Attention is all you need*. It uses four encoder–decoder layers and eight parallel attention heads. The attention has a dimension size of 512 and the feed-forward networks have a dimension size of 2048. Here the number of rows and columns corresponds to our image features extracted into a vector format, so for the InceptionV3, the feature vector size is 8 × 8 × 2048 and for the EfficientNet-B3 it is 10 × 10 × 1536. A drop out of 0.1 is used to avoid overfitting.

The network is trained using an Adam optimizer with a custom learning rate scheduler according to [36]. The loss is calculated using sparse categorical cross-entropy between real and predicted SELFIES.

### Training the models

Initially, all the models were trained using an in-house server equipped with an Nvidia V100 Tesla with 32 GB GPU, 384 GB of RAM and two Intel(R) Xeon(R) Gold 6230 CPUs. The details regarding the scaling and performance were explained in our previous publication [18]. For this work, a model with a dataset of 1 million molecules is initially trained using the same GPU equipped server. A batch size of 512 images is used to train the model, resulting in an epoch time of 29 min and 48 s, on average. For a complete convergence of the model, it took about 1 day, 5 h and 48 min on the hardware mentioned above.

On a TPU v3-8 (TPU version 3 with 8 nodes) the same model was trained with a batch size of 1024 which is distributed between 8 nodes, and it took on average 8 min and 41 s per epoch and for a complete convergence of the model, it took 8 h 41 min and 4 s. This is a reduction of 71.9% in computation time and we, therefore, decided to train all models with the TensorFlow distributed training API using the Tensor Processing Units v3-8.

### Testing the models

All the models were trained until their training loss converged, then each model was tested with a test data size of 10% of the training data. Throughout the process of selecting molecules for the test dataset, the RDKit [40] MaxMin algorithm is used to select a diverse test dataset covering the same chemical space as the training dataset.

Test dataset evaluations were performed on the GPUs. Predicted SELFIES were decoded back to SMILES and then the Tanimoto Similarity Index was calculated for the original and predicted SMILES using PubChem fingerprints, included in the CDK. The Tanimoto Similarity Index provides useful information because it makes a difference whether a wrong prediction is completely wrong or provides a result very similar to the correct molecule was used because the predictions do not always correspond to the same molecule. The Tanimoto similarity thereby provides a quantitative measure of how well the network is able to “understand” graphical chemical structure representations.

Apart from that for the predictions with the Tanimoto similarity index of 1.0, we additionally generated InChIs using the CDK to perform an isomorphism check and determined whether Tanimoto 1.0 predictions are a good proxy for structure identity.

Models trained with augmentations were tested with augmented images and with images without any augmentation.

## Results and discussion

### Computational considerations

Training large datasets such as the ones used here on deep neural networks take months even on GPUs, let alone regular CPUs. For performance measure, a dataset with 1 million molecules was trained for 50 epochs on an Nvidia Tesla V100 GPU and the same model was also trained on a TPU V3-8 (version 3 TPU with 8 nodes) and TPU V3-32 (version 3 TPU with 32 nodes).

**Fig. 4:**
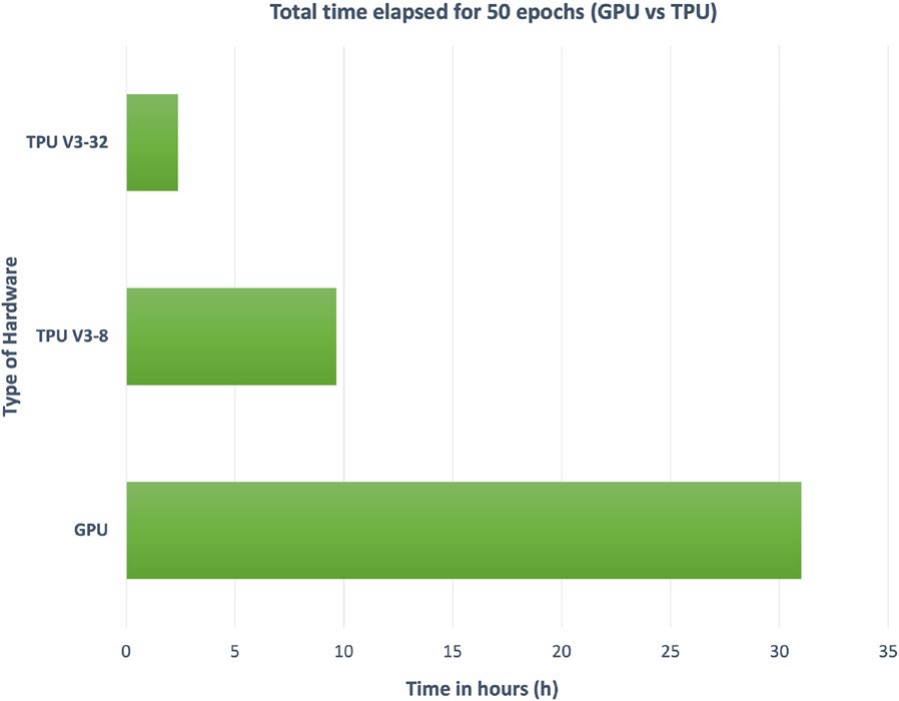
Training time comparison between a GPU and TPUs (lower is better)

Training a model on a V3-8 TPU helped by increasing training speed up to 4 times compared to a V100 GPU and by using a V3-32 TPU a 16 times faster training speed was achieved, see Fig. 4. Concerning these results and considering the costs of V3-32 TPUs, it was decided to train all the models on a V3-8 TPU.

**Fig. 5:**
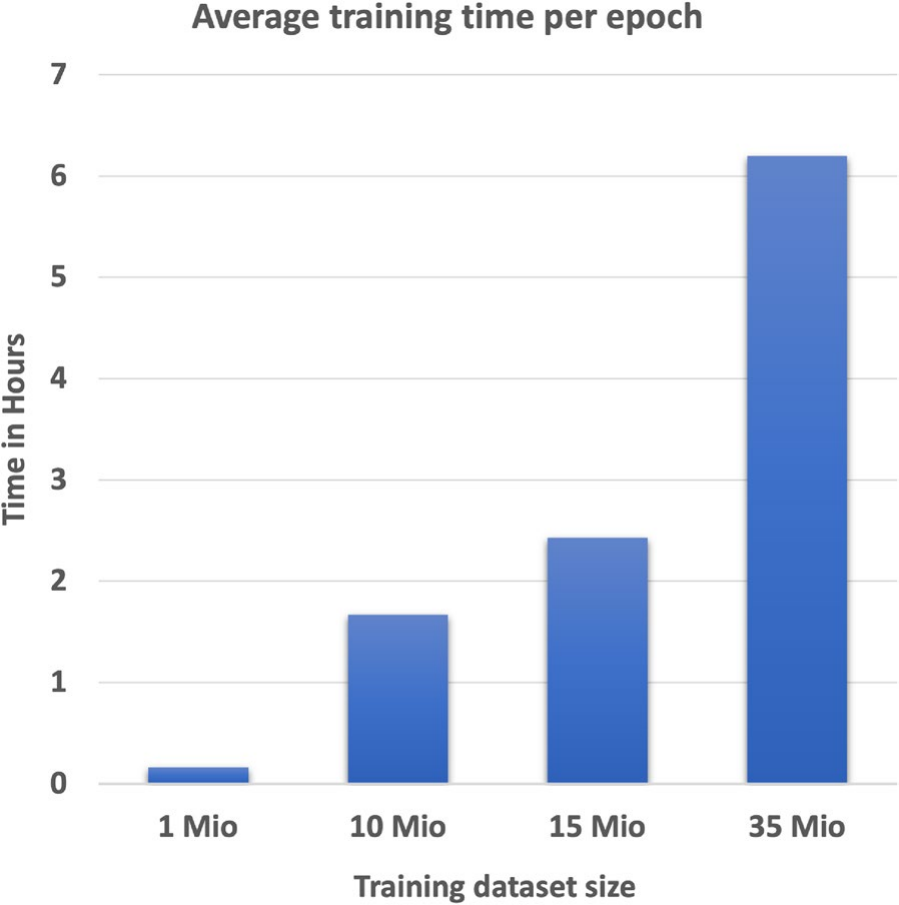
Average training time per epoch with increasing training dataset size

To evaluate if testing accuracy could be improved by increasing the training dataset size, different subsets generated using dataset 1 were trained on TPU V3-8. The maximum length of SELFIES strings stayed the same throughout the training. As shown in Fig. 5, training time increases with the increase in datasets.

It would take a considerable amount of time to examine the performance of the network using a bigger dataset. For the initial tests, Subset 1: a subset of 1 million was used, which was derived from Dataset 1. We split the dataset into 90% training data (921,600) and 10% test data (102,400) using the RDKIT MaxMin algorithm to ensure that the test data picked are diverse and resemble the training dataset.

### Image feature extraction test

Correct extraction of the image features will result in an improved overall model at the end. In our previous work, the InceptionV3 model was used for image feature extraction. InceptionV3 is a state-of-the-art image classification network. A newer network, called EfficientNet, was created to enable better classification accuracy, and the results of noisy-student training using EfficientNet [31] were better than the InceptionV3 network. The EfficientNet-B3 model was then compared to InceptionV3 while still using the same image size (299 × 299) to test whether EfficientNet-based image feature extraction would improve our models’ accuracy.

To compare the InceptionV3 feature extraction with EfficientNet-B3 feature extraction a subset of 1 million molecules was used. Using these models, the features were extracted and then used to train encoder–decoder based networks for 60 epochs until the training loss converged. The training time for the network that uses the features extracted using the InceptionV3 model was found to be shorter than the network which uses the EfficientNet-B3 model.

After training, the models were tested with a test dataset. The predicted SELFIES were retranslated into SMILES strings and the Tanimoto similarity index was calculated between original SMILES and the retranslated SMILES. Here, no errors have occurred in translating SELFIES to SMILES. Table 3 summarizes the evaluation.

**Table 3:** 1 million molecules model testing results for comparing InceptionV3 and EfficientNet-B3 feature extraction

Metrics	InceptionV3	EfficientNet-B3
Average training time per epoch	7 min 34 s	8 min 57 s
Tanimoto	0.5459	0.6345
Tanimoto 1.0	1.41%	7.03%

The Tanimoto 1.0 count indicated that the EfficientNet-B3 model led to a remarkable overall performance increase, so it was used for the entire work.

### Encoder–decoder model vs. transformer model

In our previous work [18], the encoder–decoder network was extensively explored. Meanwhile, great progress was made in transformer-based networks and the results seemed promising, so we decided to implement a transformer-based network in this work as well.

First, the transformer network was tested with InceptionV3 based image feature extraction, then it was tested using the EfficientNet-B3 based image feature extraction. The extracted image features with tokenized SELFIES were used as inputs for the transformer. For this work, the same 1 million molecules subset was used with a 90:10 split for training and testing.

The models were trained on TPU V3-8 until the training loss converged. The average time for transformer-based models was higher than the other, and the highest average training time was recorded for the EfficientNet-B3 Transformer network. Once the training was completed, the models were tested using the same test set. Table 4 summarizes the final evaluation.

**Table 4:** Comparing the encoder–decoder- and transformer-based approach with a 1 million images test dataset

Metrics	Encoder–decoder		Transformer	
	InceptionV3	EfficientNet-B3	InceptionV3	EfficientNet-B3
Average training time per epoch	7 min 34 s	8 min 57 s	8 min 33 s	9 min 27 s
Tanimoto	0.5459	0.6345	0.8764	0.9371
Tanimoto 1.0	1.41%	7.03%	55.29%	74.57%

By comparing the Tanimoto 1.0 count, the transformer-based models clearly outperformed the encoder–decoder based models.

With these results, it was decided to train all the other datasets using transformers with image features extracted using EfficientNet-B3 based image feature extraction.

### Image feature extraction comparison using EfficientNet-B3 and B7

The work described in [29] indicated that EfficientNet-B7 outperforms EfficientNet-B3 marginally by 2.7%. We, therefore, implemented EfficientNet-B7 image feature extraction and training on the extracted features. The number of parameters to train using EfficientNet-B7 (66 million parameters) compared to B3 (12 million parameters) is almost 5.5 times larger, however, which makes the network rather big and complex. Furthermore, images had to be rescaled to 600 × 600 for B7, in which the chemical structure depictions had to be magnified twice the normal scale. For B3, it is easy to use the images with a scale of 299 × 299 without any alterations.

To test these two image feature extraction methods and to see how well this helps us to achieve our main goal, a 1 million molecules image subset was used to train the transformer networks and the final models were evaluated using respective Images generated using the same test set. Table 5 summarizes the results.

**Table 5:** Comparison of evaluation of using EfficientNet-B3 and B7 for image feature extraction

Metrics	EfficientNet-B3	EfficientNet-B7
Train data size	921,600	921,600
Test data size	102,400	102,400
Train data size	0.46 TB	2.8 TB
Average training time	9 min 27 s	11 min 42 s
Tanimoto	0.9371	0.9669
Tanimoto 1.0	74.57%	84.82%

It is evident that the Image feature extraction using EfficientNet-B7 outperforms B3. We found, however, that most of the chemical structure depictions found on printed literature can easily fit the scale of 299 × 299, so to use the 600 × 600 scale the images should be upscaled. Upscaling will result in losing information which will be a major downside for this approach since the models majorly rely on the image features.

Chemical structure depictions larger than 299 × 299 square pixels can be downscaled easily to be used in our models without losing any pixel information. Thus the size of the image was decided to be 299 × 299 and the feature extraction was performed using EfficientNet-B3.

It may be possible in the future to use EfficientNet-B7 to extract image features for chemical image depictions with higher resolutions.

### The performance measure with increasing dataset size

To evaluate how the split percentage of training and test data affected the training efficiency, we use a small toy dataset (subset 1). The data was split into different sizes (see Table 6) of train and test sets using the RDKit MaxMin algorithm, and then each model was trained separately and evaluated. Table 6 summarizes the results.

**Table 6:** Results of training the subset 1 with different train and test dataset sizes

No.	Train data size	Test data size	Split	Average time per epoch	Average Tanimoto	Tanimoto 1.0 (%)
1	102,400	921,600	10|90	42.22	0.86	45.05
2	204,800	819,200	20|80	69.95	0.91	63.59
3	307,200	716,800	30|70	199.52	0.93	71.63
4	409,600	614,400	40|60	276.09	0.94	73.93
5	512,000	512,000	50|50	320.25	0.95	77.37
6	614,400	409,600	60|40	392.51	0.96	84.50
7	716,800	307,200	70|30	448.91	0.97	85.38
8	819,200	204,800	80|20	535.57	0.96	82.89
9	921,600	102,400	90|10	560.47	0.94	75.06

**Fig. 6:**
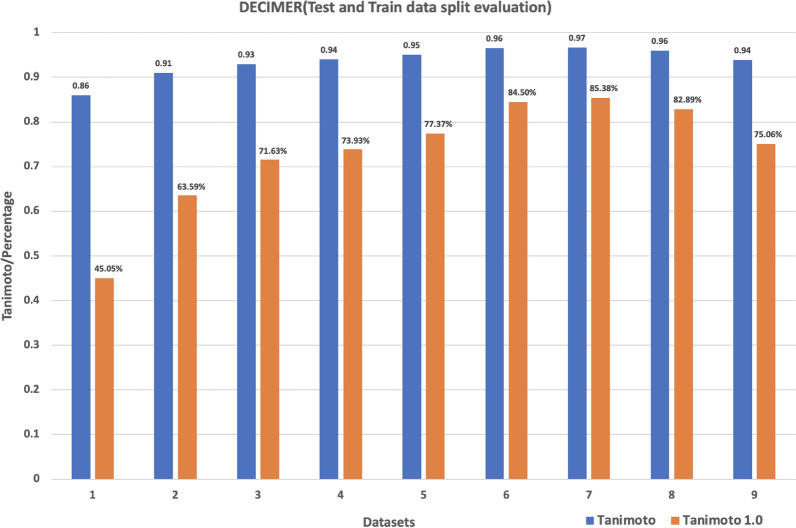
Average Tanimoto similarity indices and Tanimoto similarity 1.0 count with dataset number

Figure 6 shows that model performance increases with training dataset size. The test data performance increases up to a 70:30 split and then drops slightly, for which we have no explanation. Since we assumed that for our much larger final training data (30 Mio) it would be beneficial for the network to see as much training data as possible, we used a 90:10 split for our final training. To see how well the transformer performs with an increased number of data another subset of 10 million molecules images which was derived from the Dataset 1 (Subset 2) was utilized. The image features were extracted using the InceptionV3 based network and the EfficientNet-B3 based network. Every dataset was converted into TFRecords and moved to the Google cloud. Two different models based on these two different image feature extractions were trained. After the model completed the training they were tested using a test dataset size of 1 million molecule images of chemical structure depictions. Table 7 summarizes the results.

**Table 7:** Testing results of the models trained on 10 million molecule images of chemical structure depictions

Metrics	InceptionV3	EfficientNet-B3
Train data size	10,240,000	10,240,000
Test data size	1,024,000	1,024,000
Tanimoto	0.9310	0.9695
Tanimoto 1.0	74.52%	87.85%

Looking at the Tanimoto similarity average and the Tanimoto 1.0 count one can see that the dataset trained with the EfficientNet-B3 based image feature extraction method outperforms the InceptionV3 based method. This also was evident in the previous training with 1 million molecule images. With these results, the next set of training included only the EfficientNet-B3 based image feature extraction.

A total of four subsets were now extracted from Dataset 1, the train and test datasets were created using the RDKit MaxMin algorithm. All four datasets included the same number of tokens. All four datasets were converted into TFRecords and stored on Google Cloud Storage Buckets and used to train the models. Table 8 summarizes the overall results for different subsets.

**Table 8:** Test data results for subsets

Metrics	Subset 1	Subset 2	Subset 3	Subset 4
Train data size	921,600	10,240,000	15,360,000	35,002,240
Test data size	102,400	1,024,000	1,536,000	3,929,093
Tanimoto	0.9371	0.9691	0.9779	0.9923
Tanimoto 1.0	74.57%	87.88%	91.02%	96.47%

These results demonstrate an increasing trend of accurate predictions due to increasing data in the training datasets. In addition, with 35 million molecules training, we reached an average Tanimoto similarity of 0.99, along with a 96.47% Tanimoto 1.0 count. Because of using SELFIES as the input textual data, all of the predictions were successfully retranslated into valid molecules. An isomorphism check using InChIs was carried out in order to find out how many molecules in Tanimoto 1.0 are fully isomorphic.

InChI strings were generated using the CDK for all the predictions with a Tanimoto similarity index of 1.0 and then checked whether they are isomorphic or not by string matching.

Table 9 shows that 99% of all predictions which have Tanimoto 1.0 are structurally identical to the depicted molecule. Also with the increasing Training dataset size, the isomorphic structure count kept increasing slightly.

**Table 9:** Results of isomorphism calculations for the subsets of dataset 1

Metrics	Subset 1	Subset 2	Subset 3	Subset 4
Train data size	921,600	10,240,000	15,360,000	35,002,240
Test data size	102,400	1,024,000	1,536,000	3,929,093
Predictions with Tanimoto 1.0	74,176	899,941	1,398,028	3,790,273
Isomorphic predictions	98.63%	99.45%	99.59%	99.75%
Non-isomorphic predictions	1.37%	0.55%	0.41%	0.25%

### Analysis of the predictions with low Tanimoto similarity indices

**Fig. 7:**
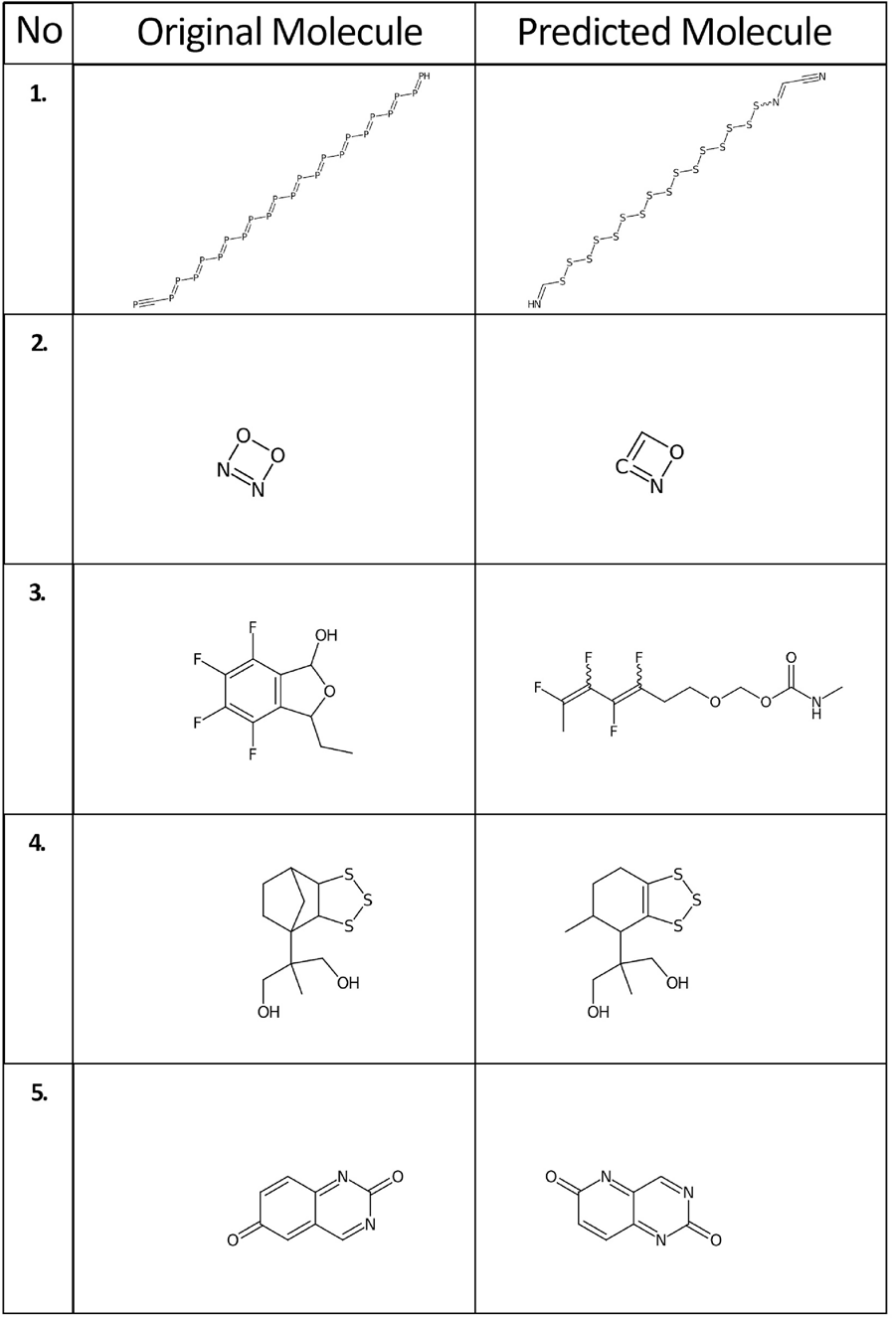
Depictions of chemical structures with lower Tanimoto similarity indices

The model trained with subset 4 was able to extract machine-readable representations of molecules depicted in the test dataset with near 100% accuracy. In order to understand why predictions with low Tanimoto scores were not predicted correctly, the following analysis was performed (Table 10; Fig. 7).

**Table 10:** Predicted SMILES with lower Tanimoto similarity indices compared with the original SMILES

No.	Original SMILES	Predicted SMILES	Tanimoto similarity index
1	P#CP=PP=PP=PP=PP=PP=PP=PP=PP=PP=PP=P	N#CC=NSSSSSSSSSSSSSSSSC=N	0
2	N1=NOO1	C=1=NOC1	0.14
3	OC1OC(C=2C(F)=C(F)C(F)=C(F)C21)C)C	O=C(OCOCCC(F)=C(F)C(F)=C(F)C)NC	0.35
4	OCC(C)(CO)C12CCC(C1)C3SSSC32	OCC(C)(CO)C1C=2SSSC2CCC1C	0.59
5	O=C1N=CC2=CC(=O)C=CC2=N1	O=C1N=CC2=NC(=O)C=CC2=N1	0.81

In most cases the network was able to interpret the skeleton of the chemical structure well. Semantically small errors such as the miss of a ring closure will lead to seemingly large errors in the eyes of a chemist, as can be seen in case 3(Fig 7).

In the majority of cases, the Tanimoto similarity was low due to the predicted SMILES,

- having one or more wrong atoms.
- missing a bond.
- having a wrong bond.
- missing an aromatic ring.

A strategy to overcome such issues could be to use multiple depictions of the same chemical structure in the training set with different rotations so that the network sees more examples of the same set of input data. Also implementing different and more image augmentation methods and training the augmented images along with the non augmented images might enable the network to see the chemical structures clearer.

### Performance of the network with training data using stereochemistry information—Dataset 2

To assess the performance of the transformer network on chemical structure depictions with stereochemistry and ions, the same dataset was used but stereochemistry and ion information were included. By including this information the unique number of tokens increased, and the molecules with the least number of tokens were removed after the calculation of the token distribution. A new dataset with 37 Mio molecules was created and split into training and test datasets using the RDKit MaxMin algorithm. This whole dataset is called Dataset-2 from now on.

By adding stereochemical information and ions, the number of unique SELFIES tokens increased from 27 to 61, almost twice the number of the tokens found on Dataset 1. From Table 11 one could see the same molecule with and without stereochemistry and how it affects the number of tokens present in the SELFIES and the depicted structure.

**Table 11:** Analysis of a molecule for with and without stereochemical information

		Molecules with stereochemical information	Molecules without stereochemical information
01	SMILES (canonical/isomric)	C1=CC2=C(C=C1C=O)C(C(O2)Br)Br	C1=CC2=C(C=C1C=O)[C@@H]([C@H](O2)Br)Br
	SELFIES	[C][=C][C][=C][Branch1_1][Branch1_3][C][=C][Ring1][Branch1_2][C][=O][C][Branch1_1][Branch2_1][C][Branch1_1][Ring2][O][Ring1][Branch2_2][Br][Br]	[C][=C][C][=C][Branch1_1][Branch1_3][C][=C][Ring1][Branch1_2][C][=O][C@@Hexpl][Branch1_1][Branch2_1][C@Hexpl][Branch1_1][Ring2][O][Ring1][Branch2_2][Br][Br]
	Number of unique SELFIES tokens	12	14
	Depicted structure		
02	SMILES (canonical/isomeric)	CC1C(=C(N(N1)C)OC2CCC=CC2)C=NO	CC1C(=C(N(N1)C)OC2CCC=CC2)/C=N/O
	SELFIES	[C][C][C][Branch2_2][Ring1][Ring2][=C][Branch1_1][Branch2_1][N][Branch1_1][Ring2][N][Ring1][Branch1_1][C][O][C][C][C][C][=C][C][Ring1][Branch1_2][C][=N][O]	[C][C][C][Branch2_2][Ring1][Ring2][=C][Branch1_1][Branch2_1][N][Branch1_1][Ring2][N][Ring1][Branch1_1][C][O][C][C][C][C][=C][C][Ring1][Branch1_2][/C][=N][/O]
	Number of unique SELFIES tokens	11	13
	Depicted Structure		

Inclusion of stereochemistry increased the amount of tokens, but also introduced new artifacts in chemical structure depictions such as wedged and dashed bonds. Including the cis/trans information reduced the amount of curly bonds in the new dataset.

**Fig. 8:**
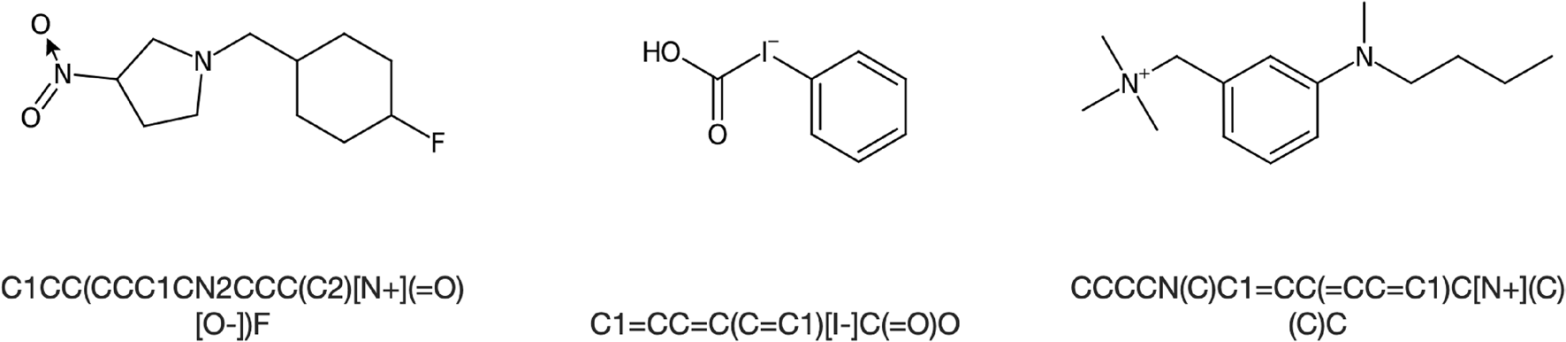
Chemical structure depictions with ions

Including the information about the ions also increased the number of tokens, also this introduced new artifacts to the chemical structure depictions such as the “+, −” signs and arrows, see Fig. 8.

Two subsets of Data Set 2 were generated, one with the 15 million training molecules plus 1.5 million test molecules and another with 33 million training molecules plus 3.7 million test molecules. TFRecords were generated from the chemical structure depictions using these datasets and moved into Google cloud storage buckets. Finally, two models were trained using these two datasets. Table 12 summarizes the results.

**Table 12:** Results on the subsets of dataset 2

Metrics	Subset 5	Subset 6
Train data size	15,360,000	33,304,320
Test data size	1,536,000	3,700,480
Tanimoto	0.9372	0.9761
Tanimoto 1.0	75.23%	89.87%

It can be seen from the results shown in Table 12 that the average Tanimoto is lower compared to dataset 1 that was seen in Table 8. The Tanimoto 1.0 count is also lower. This is mainly due to the new artefacts included in the new dataset and now the number of tokens in use also doubled. Increasing the data for the newly introduced tokens can improve the results significantly. To check how many of the predicted structures are isomorphic the InChIs were generated for the original and predicted structures and a string matching was performed as explained before, see Table 13.

**Table 13:** Results of isomorphism calculations for the subsets of dataset 2

Metrics	Subset 5	Subset 6
Train data size	15,360,000	33,304,320
Test data size	1,536,000	3,700,480
Predictions with Tanimoto 1.0	1,155,483	3,325,656
Isomorphic predictions	96.42%	98.50%
Non isomorphic predictions	3.58%	1.50%

**Fig. 9:**
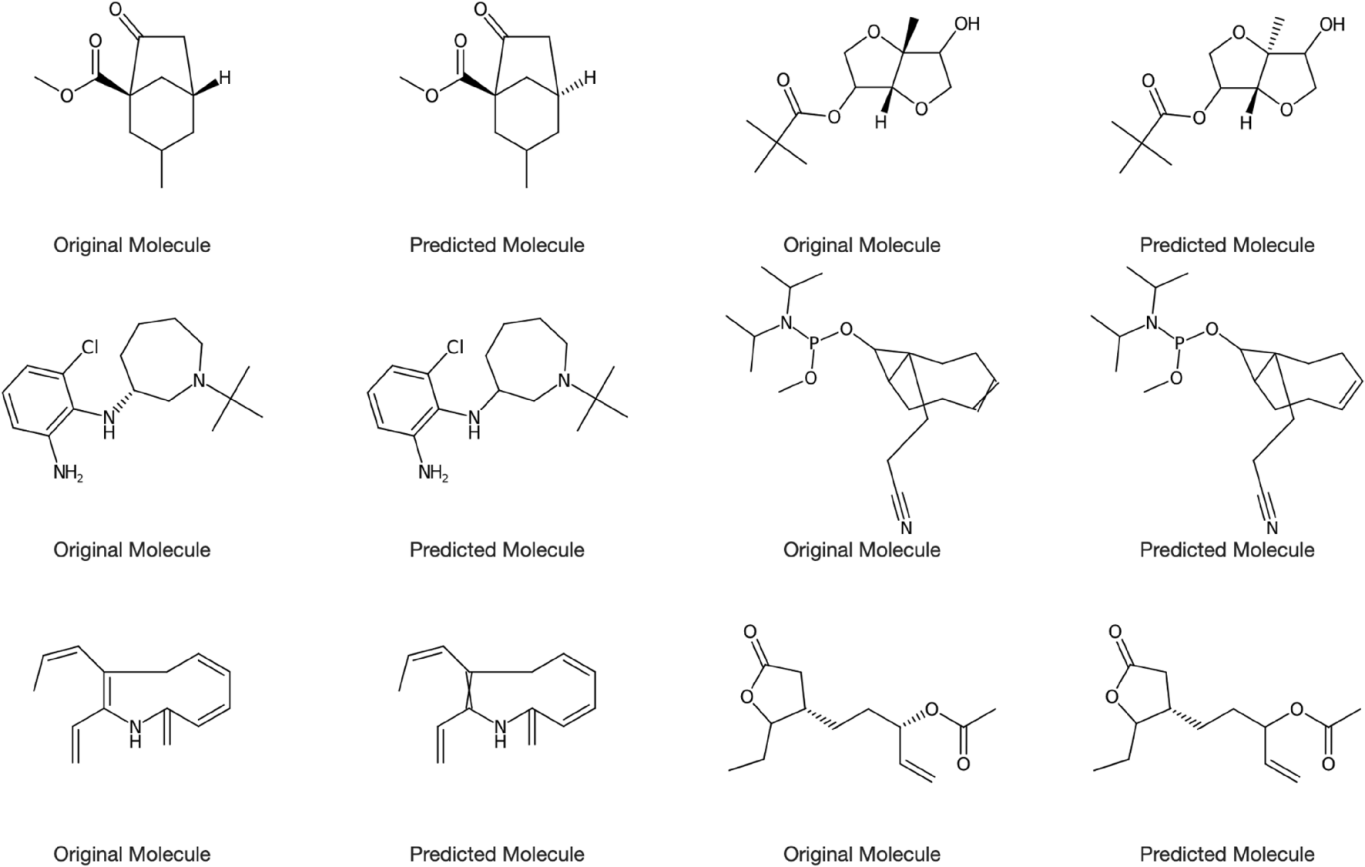
Non-isomorphic structures with Tanimoto similarity index of 1.0. The constitution is the same but predicted stereo-chemistry differs

Table 13 shows that more than 96% of the predicted SMILES are isomorphic for a training data set of 15 Mio compounds. By approximately doubling the training dataset size, the number of isomorphic structures increased to over 98%, which is similar to the results for dataset 1. We also analysed the non-isomorphic predictions to see why they had a Tanimoto similarity of 1.0. Figure 9 demonstrates that the non-isomorphic structures are identical to the original structures, except for an incorrectly predicted stereochemistry. This can very likely be improved by training the network with more molecules with stereochemistry.

Increasing the training data points will likely increase isomorphic structure predictions in general. Due to the applied ruleset, only a limited amount of data is available to work with. Therefore, the next step will be to train these models on augmented images to assess whether or not they improve overall accuracy.

### Performance of the network with training data using stereo-chemistry and image augmentation—Dataset 3

**Fig. 10:**
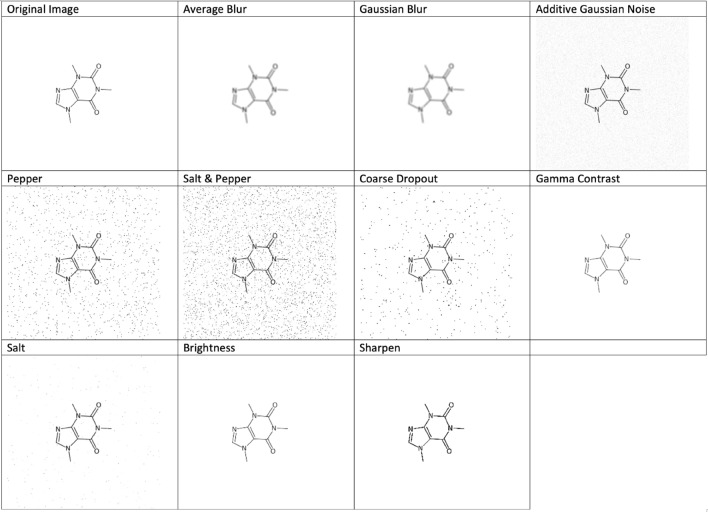
Images augmented with parameters within a given range

By applying image augmentation to dataset 2 we generated dataset 3. The resulting images look similar to Fig. 10.

The parameters were restricted to reflect the real world images, not to add extreme augmentations. The parameter is shown in Table 14 during augmentations. Here the list of parameters provided is the ones that were implemented to augment the images, for more details about the parameters and how they are implemented, we refer our readers to the imgaug documentation [41].

**Table 14:** Image augmentations and their parameters

Image augmentations	Parameters (imgaug)
Gaussian blur	0–1.8
Average blur	k = 0–3
Additive Gaussian noise	Scale = (0, 0.1 * 255)
Salt and pepper	0–0.05
Salt	0–0.05
Pepper	0–0.05
Coarse dropout	0–0.01, size percent = 0.9
Gamma contrast	0.5–2.0
Sharpen	Alpha = (0.0, 1.0), lightness = 1.0
Enhance brightness	Factor = (0.95, 1.5)

The generated dataset was then used to train two models. One model was trained from scratch using augmented images. Another model previously trained on Dataset 2 was used as the pre-trained model and then refitted with the augmented images (see Table 15). Both of them were tested on a dataset size of 4 million images, which includes 2 million images with augmentations and 2 million images without any augmentations. Table 15 summarizes the results.

**Table 15:** Results on dataset 3 and dataset 2 + 3

Metrics	Augmented dataset (3)		Pre-trained model + augmented dataset (2 + 3)	
	Non augmented test set	Augmented test set	Non augmented test set	Augmented test set
Train data size	33,304,320	33,304,320	33,304,320	33,304,320
Test data size	2,000,000	2,000,000	2,000,000	2,000,000
Tanimoto	0.9663	0.9501	0.9708	0.9521
Tanimoto 1.0	86.43%	80.26%	88.04%	80.87%
Isomorphic Predictions	97.89%	97.46%	98.15%	97.61%
Non isomorphic predictions	2.11%	2.54%	1.85%	2.39%

The first two columns of the table explain the performance of the model trained only on augmented images and tested on augmented and non-augmented images. The last two columns summarize the evaluation of the model which was previously trained on non-augmented images and refitted with dataset 2.

In refitting, we used weights from the best model previously trained on non-augmented images instead of random weights as a starting point for training. This was done to see whether using the weights from a previously trained model would improve the performance of the newly trained model trained using a similar type of data.

The above results clearly show that our models were able to retain the Tanimoto average of above 0.95 and Tanimoto 1.0 of above 80%. Also, the isomorphic results are high in all cases, and this was similar to the earlier results. The overall accuracy of these models could be improved by increasing the number of Augmented and Non-Augmented training images.

Very likely, training with more data will improve the outcome.

## Conclusion and future work

In our preliminary communication [18], we claimed that with data around 50–100 million molecule images will help us obtain a model that can predict SMILES with about 90% accuracy. Here, we have now presented a solution based on a transformer network that delivers this promise.

Using the improved EfficientNet-B3 method rather than Inception-V3 for image feature extraction helped in extracting relevant features required for network training. Through the implementation of the new transformer-based models, we've been able to improve the accuracy of our Image-to-SMILES models overall.

We have achieved an accuracy level of about 96% for chemical structure depictions using DECIMER’s new algorithm without stereochemistry training the network using 30–35 million molecules.

When the models were extended to include stereochemical information and ions, a near 90% accuracy was achieved, despite increasing the number of tokens twofold. This can be further improved by increasing the data on stereochemical information and ions. This also applies to the models trained using image augmentations. In order to improve these models, more data should be incorporated into training.

With TPUs, the models could be trained within days, and the largest model took less than 14 days to train. That means even bigger models could be trained within a month using TPUs rather than training on GPUs, which may take several months to complete. It is also cost-effective as well as energy-efficient to implement the TPU solution on the Google cloud platform rather than relying on the local hardware setup.

Our results showed that DECIMER was achieving the intended objective with synthetic data. Further steps in future will include training with more data, refining models using a variety of real-world examples and image datasets with more augmentations. Additionally, training images created by using a variety of tools will contribute to the model's improved accuracy. Ultimately, the DECIMER project aims to provide an open-source tool that is capable of performing optical chemical structure recognition (OCSR) reliably on segmented images from the scanned literature.

The DECIMER software is fully open-source and hosted on GitHub. All data and trained models are openly available.

## Data Availability

The code for DECIMER and the trained models are available at https://github.com/Kohulan/DECIMER-TPU, 10.5281/zenodo.4730515. The data is available as SMILES at: 10.5281/zenodo.4766251.

## Abbreviations

API: Application Programming Interface
CDK: Chemistry Development Kit
CNN: Convolutional Neural Network
CSR: ChemSchematicResolver
FTP: File Transfer Protocol
GB: GigaByte
GPU: Graphical Processing Unit
GRU: Gated Recurrent Unit
InChI: International Chemical Identifier
DECIMER: Deep lEarning for Chemical ImagE Recognition
MB: MegaByte
NNs: Neural Networks
OCSR: Optical Chemical Structure Recognition
OSRA: Optical Structure Recognition Application
PDF: Portable Document Format
PNG: Portable Network Graphics
RNN: Recurrent Neural Network
SDG: Structure Diagram Generator
SELFIES: Self-referencing embedded strings
SMILES: Simplified Molecular-Input Line-Entry System
TFRecord: TensorFlow Record
TPU: Tensor Processing Unit
